# SH003, A Standardized Herbal Supplement, and NK Cell Cytotoxicity in Older Adults With Recurrent Upper Respiratory Infections: A Randomized, Double‐Blind, Placebo‐Controlled Trial

**DOI:** 10.1002/fsn3.72585

**Published:** 2026-11-13

**Authors:** Chunhoo Cheon, Soobin Jang, Seong‐Gyu Ko

**Affiliations:** ^1^ Department of Preventive Medicine, College of Korean Medicine Kyung Hee University Seoul Republic of Korea

**Keywords:** herbal extract, innate immunity, natural killer cell, randomized controlled trial, SH003

## Abstract

This study evaluated the immunomodulatory efficacy and safety of SH003—a standardized herbal extract derived from *Astragalus membranaceus*, *Angelica gigas*, and *Trichosanthes kirilowii*—in adults with a mildly weakened immune system. In this single‐center, randomized, double‐blind, placebo‐controlled trial, 120 adults (predominantly older women; mean age 61.4 years; 91.7% female) with a history of recurrent upper respiratory infections were randomly assigned in a 1:1 ratio to receive either SH003 (2 g/day) or a placebo for 8 weeks. The primary outcome was the change in natural killer cell cytotoxic activity from baseline to week 8. Secondary outcomes included serum cytokine levels, systemic inflammatory markers, stress, respiratory symptoms, and safety parameters. Efficacy analyses were adjusted for baseline covariates using analysis of covariance. After 8 weeks, the SH003 group demonstrated a statistically significant increase in NK cell cytotoxicity at an effector‐to‐target ratio of 1.25:1 compared to the placebo group in the per‐protocol analysis (*p* = 0.044, ANCOVA‐adjusted). However, this finding was not replicated in the full analysis set (*p* = 0.112) or intention‐to‐treat analysis (*p* = 0.131), indicating limited robustness. No significant between‐group differences were observed for cytokine levels or respiratory symptom scores. Erythrocyte sedimentation rate was reduced in the SH003 group; this did not reach significance in the unadjusted comparison (*p* = 0.085) but became significant after ANCOVA adjustment (*p* = 0.045). No serious adverse events were reported during the trial. These findings are preliminary and exploratory; the per‐protocol result at the 1.25:1 ratio should not be interpreted as confirmatory evidence of efficacy, and replication in an independent, adequately powered trial is required.

## Graphical abstract

This randomized, placebo‐controlled trial of 120 predominantly older women with recurrent upper respiratory infections tested 8 weeks of SH003, a standardized three‐herb extract. NK cell cytotoxicity increased at one effector‐to‐target ratio in the per‐protocol analysis only, with no change in cytokines or respiratory symptoms, a reduction in erythrocyte sedimentation rate only after statistical adjustment, and no serious adverse events. These findings are preliminary and require replication.

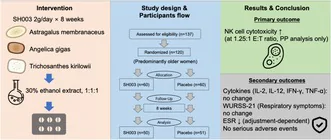

## 1 Introduction

The immune system is fundamental to maintaining physiological balance by detecting and eliminating invading pathogens, including viruses, bacteria, and other harmful agents, as well as by clearing abnormal endogenous components (Medzhitov and Janeway Jr. 1997). A well‐regulated immune response is therefore essential for sustaining human health, whereas dysregulation of this system is closely linked to increased susceptibility to infection and disease development. Broadly, immune responses are categorized into innate immunity, which provides an immediate defense through pattern recognition receptors and phagocytic cells, and adaptive immunity, which generates antigen‐specific responses and immunological memory (Garantziotis and Savani 2022; Netea et al. 2019).

In recent years, heightened exposure to environmental stressors such as air pollution, alongside global infectious disease outbreaks including the Middle East Respiratory Syndrome epidemic in 2015 and the COVID‐19 pandemic, has intensified public interest in strategies to strengthen immune defense (Lee et al. 2018). National survey data from Korea indicate that approximately half of the adult population consumes dietary supplements, with usage rates of 46.4% in men and 57.4% in women (Hwang and Hong 2023; Ministry of Agriculture 2022), reflecting growing public interest in strategies to support immune resilience and overall health. This trend highlights the need for rigorously evaluated, evidence‐based functional food ingredients. In this context, identifying plant‐derived bioactive compounds with well‐characterized immunomodulatory activity represents a key research priority in food science and nutrition.

Traditional medicinal herbs have long been investigated as sources of bioactive compounds with immunomodulatory potential. *Astragalus membranaceus* is one of the most extensively studied medicinal plants, recognized for its polysaccharide‐ and flavonoid‐rich composition that supports immune enhancement and metabolic regulation (Choi et al. 2012; Mou et al. 2011; Yin et al. 2009). Similarly, *Angelica gigas Nakai* and *Trichosanthes kirilowii Maxim* have demonstrated anti‐inflammatory and immunoregulatory activities in preclinical models, attributed to active constituents including decursin and trichosanthin, respectively (Son et al. 2009; Liu et al. 2024; Park and Park 2023; Sowndhararajan and Kim 2017; National College of Korean Medicine Publication Committee on Joint Textbook 2016; Wei et al. 2016). SH003 is a standardized herbal extract formulated from these three plants in equal proportions using 30% ethanol extraction. While SH003 has been investigated in oncological contexts (Cheon et al. 2025), its constituent herbs are food‐grade ingredients with established safety records, making SH003 a compelling candidate for evaluation as a functional dietary supplement. Preclinical studies have further demonstrated that SH003 possesses immunomodulatory properties, particularly with respect to natural killer (NK) cell activation and cytokine regulation (Han et al. 2022), providing a direct mechanistic rationale for its application in immune health supplementation. Notably, the immunomodulatory effects of SH003 were broader than those of its isolated active constituent nodakenin, which selectively stimulated a subset of cytokines, suggesting that the combined formulation may offer advantages over individual components through complementary mechanisms (Han et al. 2022).

Building on this rationale, we conducted a clinical trial to evaluate the efficacy and safety of SH003 in enhancing immune function among adults with mildly weakened immunity, defined by a history of recurrent upper respiratory infections. We hypothesized that 8‐week supplementation with SH003 would significantly enhance innate immune responses—specifically NK cell activity—compared to a placebo without causing significant adverse effects. To our knowledge, this is the first randomized, double‐blind, placebo‐controlled trial designed to rigorously validate SH003 as a safe and effective botanical supplement for immune support in adults.

## 2 Materials and Methods

### 2.1 Study Design

The study was designed to evaluate the efficacy and safety of SH003 in enhancing immune function. We conducted a single‐center, randomized, double‐blind, placebo‐controlled clinical trial at Jeonbuk National University Hospital (Republic of Korea) over an 8‐week period. The trial was conducted in accordance with the Declaration of Helsinki and the International Council for Harmonization Good Clinical Practice guidelines and was approved by the Institutional Review Board of Jeonbuk National University Hospital (IRB No. CUH 2023‐10‐049‐004) prior to participant enrollment. Although the trial protocol was finalized and submitted for registration before enrollment began, registration with the Clinical Research Information Service (KCT0009424) was completed on May 10, 2024, due to administrative processing delays, resulting in retrospective registration. Written informed consent was obtained from all participants prior to enrollment.

The study protocol was submitted for publication prior to data collection and analysis (Cheon and Ko 2025). The effector‐to‐target (E:T) ratios used in this trial (1.25:1, 2.5:1, and 5:1) differ from those listed in the published protocol paper (2.5:1, 5:1, and 10:1), which was based on a pre‐final protocol version; the ratios reported here reflect the final IRB‐approved protocol. No single E:T ratio was pre‐designated as the primary analysis ratio.

### 2.2 Participants

Eligible participants were adults aged 19–75 years who had experienced two or more upper respiratory infections, such as the common cold, pharyngitis, rhinitis, or related conditions, within the past 12 months. Participants were required to be able to comply with the study protocol and provide informed consent.

Exclusion criteria included the following: white blood cell count < 3000/μL or > 8000/μL at screening; vaccinations against influenza, shingles, pneumococcal infection, or COVID‐19 within 2 months before study initiation; body mass index < 18.5 or > 35 kg/m^2^; current use of antihypertensive medication; thyroid disorders; uncontrolled diabetes (HbA1c > 7%); clinically significant acute or chronic conditions affecting the cardiovascular, endocrine, immune, respiratory, hepatobiliary, renal and urinary, neuropsychiatric, musculoskeletal, inflammatory, hematological, oncological, or gastrointestinal systems; use of medications or health supplements affecting immune function within 1 month prior to screening; known hypersensitivity to investigational product components; use of antipsychotic medication within 3 months before screening; history or suspicion of alcohol or substance abuse; participation in another clinical trial within the past 3 months; elevated liver enzymes (aspartate aminotransferase or alanine aminotransferase > 3 times the upper limit of normal) or serum creatinine > 2.0 mg/dL; pregnancy or breastfeeding; women of childbearing potential unwilling to use adequate contraception; and any other condition judged by the investigator to make participation inappropriate.

Participants presenting with active acute illness at the time of screening were ineligible under the exclusion criterion requiring absence of clinically significant acute conditions affecting the immune or respiratory systems. The white blood cell count thresholds of 3000/μL and 8000/μL were consistent with the example eligibility criteria provided in the immune function evaluation guidelines published by the Ministry of Food and Drug Safety of Korea (Ministry of Food and Drug Safety 2020), which cite this range as an illustrative indicator of normal immune status for participants in immune function trials.

### 2.3 Randomization and Blinding

Participants who met eligibility criteria were randomly allocated in a 1:1 ratio to receive either SH003 or placebo for 8 weeks. Randomization was performed using a computer‐generated sequence prepared by an independent contract research organization, and allocation was concealed through numbered labeling of investigational products at the manufacturing site. Both investigators and participants were unaware of group assignments. The placebo tablets were identical in appearance, color, and taste to SH003 tablets.

### 2.4 Intervention

Participants in the treatment arm received SH003 (Hanpoong Pharm & Foods Co. Ltd., Korea), an herbal formulation extracted in a 1:1:1 ratio from *Astragalus membranaceus*, *Angelica gigas*, and *Trichosanthes kirilowii* with 30% ethanol. Each tablet contained 500 mg of the active formulation. Participants were instructed to take two tablets twice daily after meals (total daily dose: 2 g). The placebo contained maltodextrin and excipients identical to the active formulation and was manufactured to be visually indistinguishable. Both SH003 and placebo were produced under Korea Good Manufacturing Practice standards. Compliance was monitored via pill counting of returned medications.

The chemical composition of SH003 is controlled by the manufacturer through established specifications and testing procedures developed in accordance with the Korean Pharmacopeia. The standardized dry extract was produced by a two‐step 30% ethanol extraction at 90°C–100°C, with a drug‐to‐extract ratio of 2.68–2.96:1. Formononetin is specified as the marker compound for *Astragalus membranaceus*, and nodakenin and total decursin (decursin + decursinol angelate) are specified as marker compounds for *Angelica gigas*, consistent with the Korean Pharmacopeia monographs for these species. The same marker compounds and analytical approach have been applied consistently in prior characterizations of SH003 produced by the same manufacturer (Choi et al. 2014), supporting the reproducibility of the manufacturing process. For *Trichosanthes kirilowii*, no validated quantitative marker compound has been established in the Korean Pharmacopeia; botanical identity is confirmed by qualitative thin‐layer chromatographic identification using L‐citrulline as a reference standard in accordance with the Korean Pharmacopeia monograph for this species. Batch quality was confirmed through a 36‐month stability study. Heavy metals, residual pesticides, residual solvent, and microbial limits were tested and confirmed to comply with Korean Pharmacopeia specifications.

The chemical profile of the trial batch was further characterized by HPLC using a Waters Alliance 2690‐PDA system equipped with a T3 C18 column. The mobile phase consisted of a gradient of acetonitrile and distilled water containing 0.1% formic acid at a flow rate of 1.0 mL/min, with UV detection at 248 nm. Four major isoflavones derived from *Astragalus membranaceus*—calycosin‐7‐β‐d‐glucoside, formononetin‐7‐β‐d‐glucoside, calycosin, and formononetin—were identified and quantified by comparing their retention times and UV spectra with those of reference standard solutions.

### 2.5 Outcomes

The primary outcome was the change in NK cell activity from baseline to Week 8. NK cell cytotoxicity was assessed using a lactate dehydrogenase assay with K562 human leukemia cells as targets at E:T ratios of 1.25:1, 2.5:1, and 5:1 (CytoTox 96 Non‐Radioactive Cytotoxicity Assay Kit, Promega, WI, USA). Secondary outcomes included changes in cytokine levels (IL‐2, IL‐12, IFN‐γ, TNF‐α), inflammatory markers (C‐reactive protein [CRP], erythrocyte sedimentation rate [ESR]), stress (Global Assessment of Recent Stress [GARS]), and respiratory symptoms (Wisconsin Upper Respiratory Symptom Survey‐21 [WURSS‐21]). Safety was monitored through adverse event reporting, vital signs, and comprehensive laboratory screenings.

### 2.6 Sample Size Calculation

The sample size was calculated based on anticipated changes in NK cell activity, referencing data from a prior placebo‐controlled trial evaluating the immune‐enhancing effects of a natural product supplement using the same CytoTox 96 LDH assay platform (Ku et al. 2022). Specifically, the between‐group difference in NK cell activity at an E:T ratio of 12.5:1 in that study (treatment: 5.62% ± 8.24%; placebo: 0.95% ± 8.05%) yielded an effect size of 4.67 percentage points, which was used as the basis for power calculation. To achieve 80% power with a two‐sided *α* of 0.05, a minimum of 100 participants was required. Allowing for a 20% dropout rate, the final target sample size was 120 participants (60 per group).

### 2.7 Statistical Analysis

Efficacy analyses were conducted primarily in the per‐protocol population, supplemented by intention‐to‐treat (ITT) and full analysis set (FAS) analyses as sensitivity analyses. The ITT population included all 120 randomized participants. Missing post‐baseline values in the ITT analysis were handled using the last observation carried forward (LOCF) method, whereby the most recent available observation was substituted for missing values. The FAS comprised participants who received at least one dose of the investigational product and had at least one post‐baseline efficacy assessment, excluding those with major protocol deviations likely to affect efficacy conclusions or those with no post‐randomization data; the FAS comprised 58 participants in the SH003 group and 57 in the placebo group. The safety population included all participants who received at least one dose of study medication. Continuous variables were expressed as means with standard deviations and compared between groups using independent *t*‐tests or Mann–Whitney *U* tests, as appropriate. Within‐group changes from baseline were assessed using paired *t*‐tests or Wilcoxon signed‐rank tests. For longitudinal data such as WURSS‐21 scores, repeated measures Analysis of Variance (ANOVA) was employed to assess the differences in symptom severity and quality of life between the two groups over the 8‐weeks period. Categorical data were compared using chi‐square or Fisher's exact tests. Additionally, since a baseline imbalance was observed in BMI (*p* < 0.05), ANCOVA was performed using BMI as a covariate for all efficacy variables. For variables with statistically significant baseline imbalances between groups—specifically IL‐12 (*p* = 0.044), IFN‐γ (*p* = 0.003), and ESR (*p* = 0.048)—the corresponding baseline value was additionally included as a covariate alongside BMI, to account for the potential confounding effect of pre‐existing between‐group differences on the observed changes. A two‐sided *p*‐value < 0.05 was considered statistically significant. Statistical analyses were performed using SAS version 9.4 (SAS Institute, Cary, NC, USA). No formal correction for multiple comparisons was applied across the three E:T ratios assessed for the primary outcome.

### 2.8 Data Monitoring

Study monitoring and data management were performed by an independent contract research organization to ensure protocol compliance and accuracy of reported data. Monitoring included on‐site visits, source data verification, and review of essential study documents. All data were double entered with automated range checks and stored securely with restricted access.

## 3 Results

### 3.1 Standardization of SH003 Chemical Components

The chemical profile of the trial batch was assessed by HPLC‐PDA at 248 nm using a Waters Alliance 2690‐PDA system. Four major isoflavones derived from *Astragalus membranaceus* were identified and quantified: calycosin‐7‐β‐d‐glucoside (0.10%), formononetin‐7‐β‐d‐glucoside (0.04%), calycosin (0.05%), and formononetin (0.06%). Each peak in the SH003 chromatogram (Figure 1A) was identified by comparing its retention time and UV spectrum with those of the corresponding standard solutions (Figure 1B). The measured formononetin concentration (0.06%, equivalent to 0.6 mg/g of the dry extract) exceeded the minimum specification of 0.107 mg/g established for the dry extract, confirming that the trial batch met the required quality standard.

**FIGURE 1:**
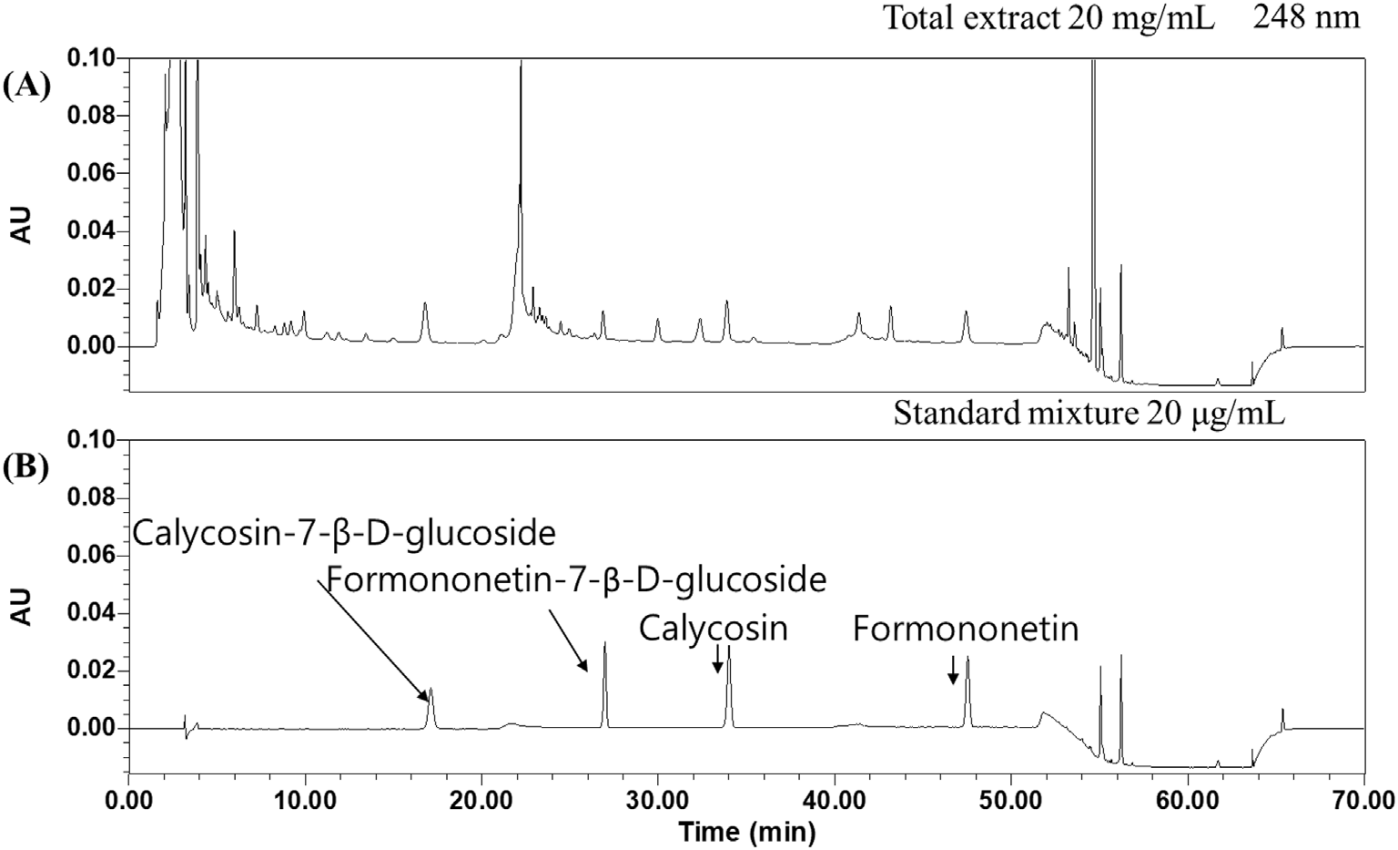
HPLC chromatograms of the (A) SH003 total extract (20 mg/mL) and (B) standard mixture (20 μg/mL) at 248 nm.

Batch analysis conducted by the manufacturer in accordance with the Korean Pharmacopeia confirmed the following species‐specific marker compound concentrations: formononetin (*Astragalus membranaceus*) at 0.107 mg/g (minimum specification); nodakenin (*Angelica gigas*) at 4.06 mg/g; and total decursin (*Angelica gigas*) at 61.54 mg/g. These values met the established specifications and were confirmed to be stable across a 36‐months stability study.

### 3.2 Participant Flow

A total of 137 individuals were screened for eligibility, of whom 17 were excluded for not meeting the inclusion criteria. The remaining 120 participants were randomly assigned to either the SH003 group (*n* = 60) or the placebo group (*n* = 60). All participants in both groups received their allocated interventions. During the 8‐weeks study period, five participants in the SH003 group and two participants in the placebo group discontinued the intervention, all due to withdrawal of consent. For the per‐protocol analysis, additional participants were excluded due to protocol violations (*n* = 4 in each group) and substantial confounding lifestyle changes, such as significant weight loss or excessive smoking (*n* = 1 in the SH003 group and *n* = 3 in the placebo group). Consequently, 50 participants in the SH003 group and 51 participants in the placebo group completed the study and were included in the final primary outcome analysis (Figure 2).

**FIGURE 2:**
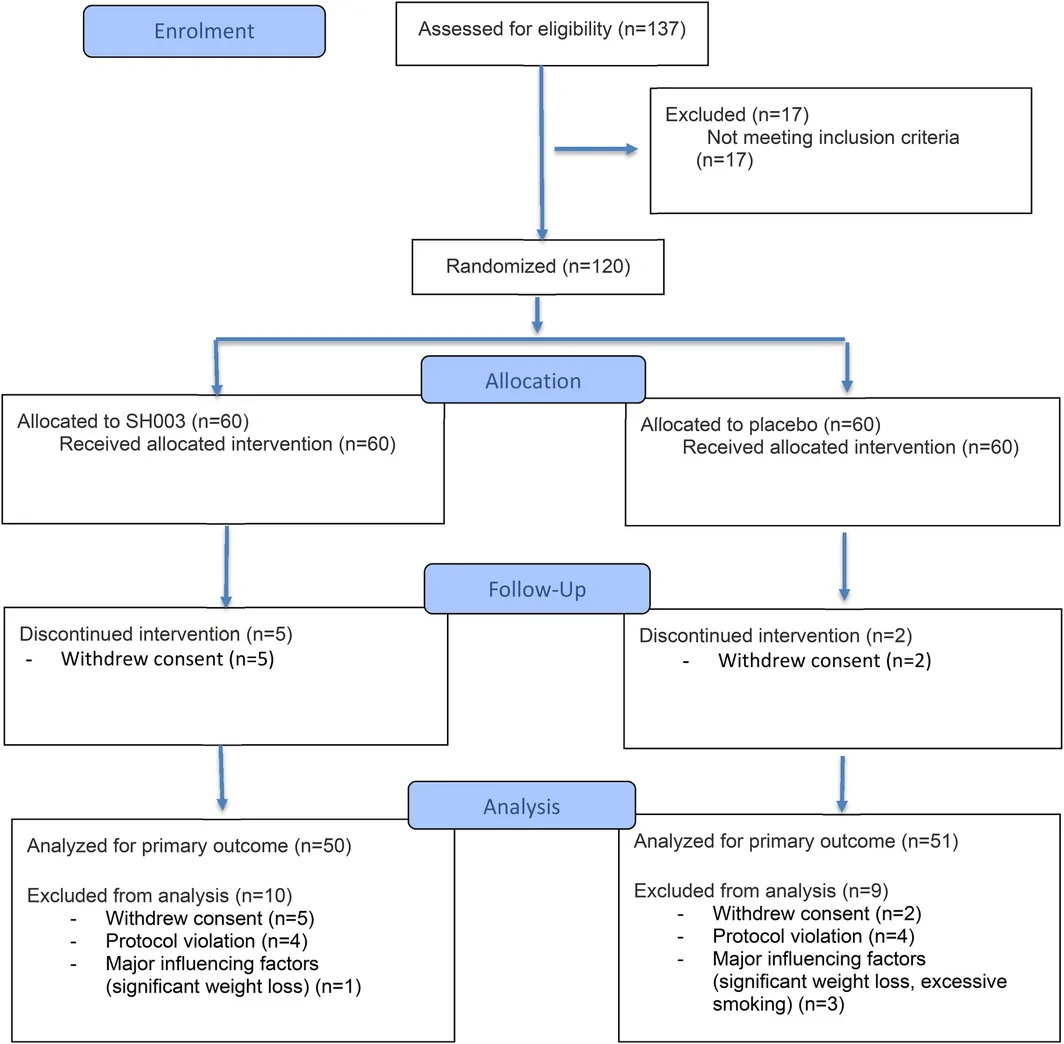
Flow diagram of patients participating in the study.

### 3.3 Baseline Characteristics of the Participants

The demographic and baseline clinical characteristics of the 120 randomized participants are summarized in Table 1. Of the total participants, 10 were male and 110 were female, with an identical sex distribution in both the SH003 and placebo groups (*n* = 5 males and *n* = 55 females per group). The mean age was 61.38 ± 5.53 years, with no significant difference between the SH003 group (60.73 ± 5.86 years) and the placebo group (62.02 ± 5.14 years). There were no significant differences between the two groups in terms of height, weight, vital signs (systolic/diastolic blood pressure and pulse rate), or HbA1c levels. Lifestyle factors, including the proportion of alcohol consumers and smokers, as well as their respective consumption amounts, also showed no statistically significant differences between the groups. However, a statistically significant difference was observed in the baseline BMI between the SH003 group (22.78 ± 2.15 kg/m^2^) and the placebo group (23.74 ± 2.38 kg/m^2^, *p* = 0.022). To account for this baseline imbalance, BMI was included as a covariate in the subsequent statistical analyses for efficacy to ensure the validity of the results. Overall, except for BMI, the randomization process effectively balanced the baseline characteristics between the two intervention groups.

**TABLE 1:** Baseline characteristics of participants.

	SH003 group	Placebo group	*p*
Sex (M/F) (*n*, %)	5 (8.3)/55 (91.7)	5 (8.3)/55 (91.7)	0.999
Age (years)	60.73 ± 5.86	62.02 ± 5.14	0.205
Height (cm)	159.70 ± 5.72	157.90 ± 5.53	0.082
Weight (kg)	58.20 ± 7.10	59.27 ± 7.27	0.415
BMI (kg/m^2^)	22.78 ± 2.15	23.74 ± 2.38	0.022
SBP (mmHg)	122.98 ± 11.50	123.35 ± 12.80	0.869
DBP (mmHg)	72.97 ± 8.88	71.57 ± 10.84	0.441
Pulse (BPM)	76.70 ± 9.80	73.60 ± 9.23	0.077
HbA1c (%)	5.63 ± 0.32	5.65 ± 0.27	0.761
Alcohol (Y/N) (*n*, %)	14 (23.3)/46 (76.7)	10 (16.7)/50 (83.3)	0.361
Amount alcohol (g)	28.43 ± 50.81	18.00 ± 24.62	0.513
Smoking (Y/N) (*n*, %)	1 (1.7)/59 (98.3)	2 (3.3)/58 (96.7)	0.999

### 3.4 Primary Outcome: Natural Killer Cell Activity

The primary efficacy of SH003 on immune function was evaluated by measuring changes in NK cell activity from baseline to Week 8. The results are summarized in Table 2. At an E:T ratio of 1.25:1, the SH003 group showed a significant increase in NK cell activity compared to baseline (14.32% ± 7.28% vs. 12.51% ± 7.97%, *p* = 0.008), whereas no such increase was observed in the placebo group (*p* = 0.883). Consequently, the difference in the change of NK cell activity between the two groups was statistically significant (*p* = 0.030). Even after adjusting for baseline BMI as a covariate, the superiority of SH003 over the placebo remained statistically significant at the 1.25:1 ratio (*p* = 0.044). At an E:T ratio of 2.5:1, the SH003 group demonstrated a significant within‐group improvement compared to baseline (23.31% ± 8.27% vs. 21.30% ± 10.50%, *p* = 0.046). Although the between‐group difference did not reach statistical significance, ANCOVA adjusting for BMI showed a numerically higher NK cell activity in the SH003 group compared to the placebo group. At the 5:1 E:T ratio, NK cell activity at Week 8 was higher in the SH003 group without reaching statistical significance (*p* = 0.062).

**TABLE 2:** Changes in natural killer cell activity from baseline to Week 8.

		SH003 group (*n* = 50)	Placebo group (*n* = 51)	*p*
1.25:1	Baseline	12.51 ± 7.97	12.63 ± 6.68	0.613^b^
	Week 8	14.32 ± 7.28^a^	12.65 ± 6.51	
	Change	1.81 ± 5.81	0.03 ± 4.25	0.030^b^/0.044^c^
2.5:1	Baseline	21.30 ± 10.50	21.29 ± 9.94	0.778^b^
	Week 8	23.31 ± 8.27^a^	21.60 ± 9.18	
	Change	2.01 ± 7.07	0.32 ± 5.66	0.128^b^/0.081^c^
5:1	Baseline	32.20 ± 11.60	30.53 ± 10.39	0.446^b^
	Week 8	34.16 ± 9.41	31.48 ± 10.27	
	Change	1.96 ± 7.25	0.95 ± 6.13	0.453^b^/0.336^c^

^a^ *p* < 0.05 versus baseline within the group by paired *t*‐test.

^b^ Analyzed by independent *t*‐test.

^c^ Analyzed by ANCOVA with the BMI as a covariate.

To assess the robustness of the per‐protocol findings, sensitivity analyses were conducted in the FAS (SH003 *n* = 58, placebo *n* = 57) and the ITT population (*n* = 120), in which missing post‐baseline values were imputed by last observation carried forward (Tables 3 and 4). In both the FAS and ITT analyses, no statistically significant between‐group differences in NK cell cytotoxicity were observed at any E:T ratio after ANCOVA adjustment (FAS: 1.25:1 *p* = 0.112, 2.5:1 *p* = 0.463, 5:1 *p* = 0.987; ITT: 1.25:1 *p* = 0.131, 2.5:1 *p* = 0.600, 5:1 *p* = 0.970), in contrast to the per‐protocol finding at the 1.25:1 ratio.

**TABLE 3:** Changes in NK cell cytotoxicity from baseline to Week 8: Full analysis set (*n* = 115).

		SH003 group (*n* = 58)	Placebo group (*n* = 57)	*p*
1.25:1	Baseline	12.86 ± 8.00	12.88 ± 6.59	0.632^b^
	Week 8	14.26 ± 7.24^a^	13.11 ± 6.72	
	Change	1.41 ± 5.53	0.22 ± 4.48	0.130^b^/0.112^c^
2.5:1	Baseline	21.68 ± 10.57	21.38 ± 9.94	0.918^b^
	Week 8	23.09 ± 8.47	22.39 ± 9.52	
	Change	1.41 ± 6.81	1.01 ± 6.08	0.619^b^/0.463^c^
5:1	Baseline	32.13 ± 11.47	30.61 ± 10.34	0.457^b^
	Week 8	33.75 ± 9.84	32.54 ± 10.44	
	Change	1.62 ± 7.14	1.93 ± 6.83^a^	0.814^b^/0.987^c^

^a^ *p* < 0.05 versus baseline within the group by paired *t*‐test.

^b^ Analyzed by independent *t*‐test.

^c^ Analyzed by ANCOVA with the BMI as a covariate.

**TABLE 4:** Changes in NK cell cytotoxicity from baseline to Week 8: Intention‐to‐treat analysis (*n* = 120).

		SH003 group (*n* = 60)	Placebo group (*n* = 60)	*p*
1.25:1	Baseline	12.71 ± 7.94	12.86 ± 6.44	0.911^b^
	Week 8	14.04 ± 7.28^a^	13.11 ± 6.57	
	Change	1.33 ± 5.45	0.24 ± 4.37	0.231^b^/0.131^c^
2.5:1	Baseline	21.63 ± 10.54	21.48 ± 9.73	0.937^b^
	Week 8	22.84 ± 8.82	22.47 ± 9.32	
	Change	1.21 ± 6.84	0.99 ± 5.93	0.852^b^/0.600^c^
5:1	Baseline	31.94 ± 11.58	30.56 ± 10.10	0.489^b^
	Week 8	33.46 ± 10.16	32.55 ± 10.18	
	Change	1.52 ± 7.04	1.99 ± 6.73^a^	0.712^b^/0.970^c^

^a^ *p* < 0.05 versus baseline within the group by paired *t*‐test.

^b^ Analyzed by independent *t*‐test.

^c^ Analyzed by ANCOVA with the BMI as a covariate.

### 3.5 Secondary Outcomes: Cytokine Levels

The effects of SH003 on cytokine levels, including IL‐2, IL‐12, IFN‐γ, and TNF‐α, were evaluated as secondary endpoints. The results are summarized in Table 5. At baseline, statistically significant differences between groups were observed in IL‐12 (7.80 ± 5.77 vs. 5.74 ± 3.77 pg/mL, *p* = 0.044) and IFN‐γ (76.98 ± 49.41 vs. 52.53 ± 32.22 pg/mL, *p* = 0.003), with the SH003 group showing higher initial values in both cytokines. Given these imbalances, all between‐group comparisons for cytokine outcomes were conducted using ANCOVA with both BMI and corresponding baseline values as covariates. There were no statistically significant differences between the SH003 and placebo groups in the change of any measured cytokines from baseline to Week 8. For IL‐2, both groups showed a modest decrease after 8 weeks, with no significant between‐group difference after BMI adjustment. For IL‐12 and IFN‐γ, the placebo group demonstrated a more pronounced within‐group decrease (*p* < 0.01), but ANCOVA‐adjusted between‐group comparisons remained non‐significant. TNF‐α levels remained stable in both groups throughout the study period.

**TABLE 5:** Changes in serum cytokine levels from baseline to Week 8.

		SH003 group (*n* = 50)	Placebo group (*n* = 51)	*p*
IL‐2	Baseline	6.79 ± 5.82	5.57 ± 3.11	0.600^b^
	Week 8	6.45 ± 5.79	5.29 ± 2.96^a^	
	Change	−0.34 ± 2.09	−0.28 ± 0.58	0.424^b^/0.612^c^
IL‐12	Baseline	7.80 ± 5.77	5.74 ± 3.77	0.044^b^
	Week 8	7.54 ± 5.99	5.36 ± 3.81^a^	
	Change	−0.26 ± 1.54	−0.37 ± 1.36	0.999^b^/0.883^d^
IFN‐γ	Baseline	76.98 ± 49.41	52.53 ± 32.22	0.003^b^
	Week 8	74.42 ± 50.01	50.44 ± 34.20^a^	
	Change	−2.56 ± 13.19	−2.10 ± 8.96	0.659^b^/0.437^d^
TNF‐α	Baseline	16.72 ± 5.15	15.36 ± 4.22	0.197^b^
	Week 8	16.20 ± 5.10	15.01 ± 4.61	
	Change	−0.52 ± 2.39	−0.35 ± 2.00	0.992^b^/0.449^c^

*Note:* Values are presented as mean ± SD.

^a^ *p* < 0.05 versus baseline within the group by paired *t*‐test.

^b^ Analyzed by independent *t*‐test.

^c^ Analyzed by ANCOVA with the BMI as a covariate.

^d^ Analyzed by ANCOVA with the BMI and baseline as covariates.

### 3.6 Secondary Outcomes: Respiratory Symptoms (WURSS‐21)

The WURSS‐21 was utilized to evaluate changes in upper respiratory infection symptoms and their impact on quality of life from baseline to Week 8. The results are summarized in Table 6. There were no statistically significant differences between the intervention and placebo groups in the changes of total WURSS‐21 scores, including symptom severity and quality of life sub‐scores, at Week 8. Furthermore, no significant differences were observed between the groups in the global assessment of respiratory symptoms during the study period.

**TABLE 6:** WURSS‐21 scores at baseline and Week 8.

		SH003 group (*n* = 50)	Placebo group (*n* = 51)	*p*
Symptom severity score	Baseline	5.76 ± 8.43	4.88 ± 7.38	
	Week 8	3.62 ± 5.57	4.37 ± 8.15	
	Change	−2.14 ± 9.01	−0.51 ± 6.53	0.521^a^/0.510^b^
Quality of life score	Baseline	3.56 ± 8.83	1.80 ± 5.02	
	Week 8	1.20 ± 2.32	1.63 ± 4.88	
	Change	−2.36 ± 8.78	−0.18 ± 2.36	0.151^a^/0.113^b^
Total WURSS‐21 score	Baseline	9.32 ± 16.92	6.69 ± 11.88	
	Week 8	4.82 ± 6.89	6.00 ± 12.62	
	Change	−4.50 ± 16.99	−0.69 ± 8.36	0.317^a^/0.290^b^

*Note:* Values are presented as mean ± SD.

^a^ Analyzed by repeated measures ANOVA between groups.

^b^ Analyzed by ANCOVA with the BMI as a covariate.

### 3.7 Secondary Outcomes: Inflammatory Markers and Perceived Stress

The levels of systemic inflammatory markers and perceived stress were assessed to evaluate the broader immunomodulatory effects of the intervention. The results are summarized in Table 7. Regarding inflammatory markers, CRP levels did not show significant differences between the two groups. However, a statistically significant difference in baseline ESR was observed between the two groups (SH003: 22.78 ± 12.57 vs. placebo: 18.08 ± 11.73 mm/h, *p* = 0.048). Within the SH003 group, the ESR significantly decreased to 18.50 ± 10.44 mm/h at week 8 (*p* = 0.003). Although the raw between‐group comparison did not reach statistical significance (*p* = 0.085), the reduction in ESR became statistically significant when using ANCOVA to account for baseline BMI and baseline ESR as covariates (*p* = 0.045). For the assessment of psychological stress, there were no statistically significant differences in the total GARS scores between the SH003 and placebo groups from baseline to Week 8. Both groups maintained stable stress levels throughout the study period without significant inter‐group variance.

**TABLE 7:** Changes in inflammatory markers and perceived stress scale scores at baseline and Week 8.

		SH003 group (*n* = 50)	Placebo group (*n* = 51)	*p*
CRP	Baseline	0.95 ± 1.63	0.84 ± 0.87	0.358^b^
	Week 8	0.61 ± 0.58	0.75 ± 0.89	
	Change	−0.34 ± 1.61	−0.09 ± 1.25	0.894^b^/0.381^c^
ESR	Baseline	22.78 ± 12.57	18.08 ± 11.73	0.048^b^
	Week 8	18.50 ± 10.44^a^	17.00 ± 11.51	
	Change	−4.28 ± 9.14	−1.08 ± 7.65	0.085^b^/0.045^d^
GARS	Baseline	7.48 ± 7.82	8.33 ± 7.00	0.565^b^
	Week 8	7.28 ± 10.75	6.69 ± 7.26	
	Change	−0.20 ± 6.70	−1.65 ± 7.65	0.315^b^/0.386^c^

*Note:* Values are presented as mean ± SD.

Abbreviations: CRP, C‐reactive protein; ESR, erythrocyte sedimentation rate; GARS, global assessment of recent stress.

^a^ *p* < 0.05 versus baseline within the group by paired *t*‐test.

^b^ By independent *t*‐test.

^c^ Analyzed by ANCOVA with the BMI as a covariate.

^d^ Analyzed by ANCOVA with the BMI and baseline as a covariate.

### 3.8 Safety Evaluation

Safety was evaluated in all 120 randomized participants who received at least one dose of the study product (safety population: SH003, *n* = 60; placebo, *n* = 60). Over the 8‐week study period, a total of 21 mild‐to‐moderate adverse events (AEs) were reported in 18 participants (10 events in the SH003 group and 11 events in the placebo group), with no statistically significant difference in AE incidence between the two groups. Reported AEs included common cold (*n* = 10), constipation (*n* = 2), dyspepsia (*n* = 2), headache (*n* = 2), generalized sensation of heat (*n* = 1), localized pruritus (*n* = 1), ankle pain (*n* = 1), elbow ligament rupture (*n* = 1), and pneumonia (*n* = 1). Only one mild event (localized pruritus in the placebo group) was assessed as possibly related to the study product, while all remaining 20 events were determined to be unrelated or definitely unrelated. No serious adverse events or severe adverse reactions occurred during the trial.

Laboratory assessments, including hematology, blood biochemistry, and urinalysis, confirmed the safety of SH003. Neutrophil counts showed a statistically significant within‐group increase in the placebo group (*p* = 0.034), whereas those in the SH003 group remained stable, resulting in a significant between‐group difference (*p* = 0.043). However, all values in both groups remained well within normal physiological reference ranges, and this change was not considered clinically meaningful. Minor within‐group fluctuations in systolic blood pressure and pulse rate were observed in the placebo group (*p* < 0.05), whereas no significant changes were noted in the SH003 group. No significant between‐group differences were found in vital signs, and all values remained within clinically acceptable ranges. Body weight and BMI showed no significant changes within or between groups throughout the study. Overall, these findings demonstrate that 8‐week oral supplementation with SH003 is safe and well tolerated in adults, supporting its suitability as a dietary supplement ingredient.

## 4 Discussion

This study is the first randomized, double‐blind, placebo‐controlled trial to evaluate the immune‐enhancing effects of SH003 in a general population with mildly weakened immune systems. Our findings extend the evidence base for SH003 beyond its oncological applications, demonstrating its potential as a functional dietary supplement capable of modulating key immune parameters in otherwise healthy individuals with mildly impaired immune function.

The primary finding of this trial was a significant increase in NK cell activity in the SH003 group compared to the placebo group, particularly at an E:T ratio of 1.25:1. NK cells play a key role in the innate immune response, providing a rapid defense against viral infections and transformed cells. The enhancement of NK cell cytotoxicity suggests that SH003 may strengthen early‐stage immune surveillance. While previous in vitro studies reported the potential of SH003 to stimulate NK cell activity (Han et al. 2022), this trial provides initial clinical evidence suggesting that SH003 may stimulate NK cell cytotoxicity in humans, though the robustness of this finding warrants careful consideration. It is important to note, however, that this isolated finding at a single E:T ratio was not accompanied by significant changes in systemic cytokine levels or clinical respiratory symptom scores and was not replicated in sensitivity analyses. SH003 therefore cannot be characterized as a broad systemic immunomodulator or clinical preventive agent on the basis of the present data.

Interestingly, SH003 also demonstrated a significant reduction in the ESR compared to the placebo group after adjusting for baseline BMI and initial values. ESR is a non‐specific marker of systemic inflammation. This result, alongside the stable levels of CRP, suggests that SH003 may exert a mild anti‐inflammatory effect while simultaneously enhancing immune cell activity. Such dual modulation—stimulating effector cells while regulating inflammatory markers—is a characteristic feature of several herbal compounds used in traditional medicine for maintaining homeostasis. Furthermore, a recent study demonstrated that SH003 alleviates acute lung injury by suppressing inflammasome activation and neutrophil extracellular trap formation, further supporting its role in preventing excessive inflammatory damage (Kim et al. 2024).

The absence of significant between‐group differences in circulating cytokine levels warrants careful interpretation given the baseline imbalances in IL‐12 and IFN‐γ. In particular, the SH003 group entered the trial with markedly higher IFN‐γ values (76.98 vs. 52.53 pg/mL), raising the question of whether a ceiling effect may have dampened the detectable response in that arm. Although ANCOVA adjustments were applied to account for both BMI and baseline cytokine values, the biological variability in these measurements—IFN‐γ especially, given its sensitivity to intercurrent infections and diurnal fluctuation—limits the conclusions that can be drawn from a single post‐intervention timepoint. Future trials should consider repeated cytokine sampling at intermediate timepoints to better characterize the trajectory of immune modulation.

The concurrent enhancement of NK cell cytotoxicity and reduction in ESR—two mechanistically distinct outcomes—points toward a pluripotent immunomodulatory profile that is consistent with the multi‐constituent nature of SH003. Among the identified isoflavones, calycosin and its glucoside C7G have been suggested in preclinical models to modulate NF‐κB signaling and CD8+ T‐cell activity (Zhang et al. 2019; Liu et al. 2026), providing a speculative mechanistic rationale for the anti‐inflammatory signal captured by ESR in the present trial. Formononetin has been shown to remodel the tumor immune microenvironment and modulate immune cell activity in single‐cell transcriptomic analyses of murine osteosarcoma models (Liu et al. 2025), suggesting a potential role in immune regulation that may extend to non‐oncological contexts. Additionally, formononetin may contribute to systemic stabilization by suppressing inflammatory signaling through MDM2/HIF1α pathways (Lan et al. 2024; Han et al. 2023). However, the relative contribution of each constituent in vivo cannot be determined from the present trial. The mechanistic pathways discussed above should be regarded as strictly hypothesis‐generating; definitive attribution requires future studies employing isolated compounds or rigorous pharmacokinetic‐pharmacodynamic profiling.

The lack of between‐group difference in WURSS‐21 scores deserves scrutiny beyond a straightforward null interpretation, given the design constraints of the instrument in this setting. The absence of significant improvement in WURSS‐21 scores is not straightforwardly interpretable as a null effect on respiratory health. The WURSS‐21 was developed and validated for symptom monitoring during active upper respiratory infection episodes, and whether it retains adequate responsiveness outside of active infection episodes remains unclear. In this trial, baseline total WURSS‐21 scores averaged below 10 in both groups, indicating that most participants were not experiencing active symptoms at either assessment point. Under these conditions, further score reduction was arithmetically constrained and clinically ambiguous. The biological improvements observed in NK cell activity and ESR suggest that physiological changes may precede any subjectively perceptible shift in symptom burden, particularly within an 8‐week observation window in a predominantly asymptomatic cohort. Capturing this type of effect would require instruments and timeframes better suited to a preventive design—such as prospective illness diaries or structured monthly symptom logs that track infection incidence and episode duration over a longer follow‐up period.

It should also be noted that the study population was predominantly female (91.7%), reflecting the demographic profile of supplement consumers in Korea but limiting the extent to which findings can be generalized to men. Sex‐related variation in NK cell activity—including differences in basal cytotoxic activity and hormonal responsiveness—is well documented (Al‐Attar et al. 2016), and whether the observed response would be reproduced in a more balanced cohort warrants examination in future multicenter studies.

The interpretation of the primary finding requires consideration of its consistency across analysis populations. The per‐protocol finding at the 1.25:1 ratio was not replicated in either the FAS or ITT sensitivity analysis, indicating that the result is sensitive to the composition of the analysis population. The 19 participants excluded from the per‐protocol set—including those removed for post‐randomization lifestyle changes—may have differed systematically from those retained, introducing the possibility of selection bias. This inconsistency across analysis populations substantially limits the inferential weight of the primary finding and highlights the need for confirmation in an independent trial.

This study has several limitations that should be considered when interpreting its findings. First, the single‐center design limits the generalizability of our results to broader and more ethnically diverse populations. Second, the study population was disproportionately female (91.7%) and consisted predominantly of older adults (mean age 61.4 years), which substantially restricts the applicability of findings to men, younger adults, and populations with different sex hormone profiles. Given that NK cell activity is known to vary by sex and age, the results of this trial should not be generalized to the broader adult population. Third, the WURSS‐21, though widely used for upper respiratory symptom assessment, was designed for use during active infection episodes. In this trial, baseline total WURSS‐21 scores were below 10 in both groups (SH003: 9.32 ± 16.92; placebo: 6.69 ± 11.88), indicating that most participants were asymptomatic at both assessment points. Under these conditions, a floor effect substantially limited the sensitivity of the WURSS‐21 to detect any treatment‐related symptom improvement. Fourth, the 8‐week follow‐up period may be insufficient to observe clinically meaningful changes in infection incidence or adaptive immune parameters. Fifth, significant baseline imbalances in IFN‐γ (*p* = 0.003) and IL‐12 (*p* = 0.044) between groups—despite randomization—are particularly noteworthy given the magnitude of the IFN‐γ difference (76.98 vs. 52.53 pg/mL) and the biological sensitivity of these cytokines to intercurrent infections and individual variation. Although ANCOVA incorporating both BMI and the corresponding baseline value was applied to cytokine comparisons with significant baseline imbalances, residual biological heterogeneity may not be fully eliminated by statistical adjustment alone, and caution is warranted in interpreting the null cytokine findings. Longer trials with repeated biological sampling and a more balanced participant pool would be needed to confirm and extend these findings. Sixth, the published protocol paper listed E:T ratios of 2.5:1, 5:1, and 10:1, which differ from the 1.25:1, 2.5:1, and 5:1 ratios specified in the final IRB‐approved protocol and used in this trial. No single E:T ratio was pre‐designated as the primary analysis ratio, and no formal correction for multiple comparisons was applied; applying a Bonferroni correction would require *p* < 0.017, a threshold the observed *p*‐value of 0.044 does not meet. Statistical significance was restricted strictly to the 1.25:1 ratio and was absent at 2.5:1 and 5:1, which, together with the null findings in secondary systemic cytokines, limits the biological plausibility of the primary outcome. Additionally, the observed between‐group difference at the 1.25:1 ratio (approximately 1.78 percentage points) was substantially smaller than the 4.67 percentage point difference assumed for power calculation, indicating the trial was underpowered for the effect size actually observed. The finding at the 1.25:1 ratio should therefore be regarded as preliminary and hypothesis‐generating. Seventh, the statistically significant between‐group difference in NK cell cytotoxicity observed in the per‐protocol analysis was not replicated in the full analysis set or intention‐to‐treat analysis. This inconsistency across analysis populations suggests that the selective exclusion of 19 participants from the per‐protocol set may have influenced the primary finding and substantially limits the confidence with which efficacy conclusions can be drawn from the present data. Additionally, although the trial protocol was finalized and submitted for registration prior to enrollment, registration with the Clinical Research Information Service was completed retrospectively (KCT0009424; May 10, 2024) due to administrative processing delays. This represents a deviation from prospective registration standards and should be considered when interpreting the credibility of the pre‐specified outcomes.

Despite these limitations, this study adheres to the functional food evaluation guidelines established by the Ministry of Food and Drug Safety (2020) and provides a foundation for the development of SH003 as an evidence‐based botanical dietary supplement for immune health support.

In conclusion, this study provides preliminary and exploratory evidence that 8‐week supplementation with SH003 was associated with a statistically significant increase in NK cell cytotoxicity at the 1.25:1 E:T ratio in a per‐protocol analysis of older adults, predominantly women, with a history of recurrent upper respiratory infections. However, this finding was not replicated in sensitivity analyses using the full analysis set or intention‐to‐treat population, and should not be interpreted as confirmatory evidence of efficacy of SH003 on immune function. The concurrent reduction in erythrocyte sedimentation rate, the absence of serious adverse events, and the acceptable safety profile support the feasibility of SH003 as a candidate for further investigation. Whether SH003 can meaningfully enhance innate immune responses requires confirmation in an independent, adequately powered randomized trial with a pre‐registered primary E:T ratio and formal correction for multiple comparisons.

## Author Contributions

**Chunhoo Cheon:** conceptualization, formal analysis, writing – original draft, writing – review and editing. **Soobin Jang:** methodology, writing – review and editing. **Seong‐Gyu Ko:** supervision, funding acquisition, writing – review and editing.

## Funding

This work was supported by a grant from Kyung Hee University in 2024 (KHU‐20241067), National Research Foundation of Korea, RS‐2020‐NR049559, and Ministry of SMEs and Startups, S3302137.

## Ethics Statement

The study was approved by the Institutional Review Board of Jeonbuk National University Hospital, Republic of Korea (IRB No. CUH 2023‐10‐049‐004).

## Consent

Written informed consent was obtained from all study participants.

## Conflicts of Interest

The authors declare no conflicts of interest.

## Data Availability

The data that support the findings of this study are available on request from the corresponding author. The data are not publicly available due to privacy or ethical restrictions.
